# Vegetarian, Gluten-Free, and Energy Restricted Diets in Female Athletes

**DOI:** 10.3390/sports4040050

**Published:** 2016-10-21

**Authors:** Lynn Cialdella-Kam, Danielle Kulpins, Melinda M. Manore

**Affiliations:** 1 School of Medicine, Department of Nutrition, Case Western Reserve University, WG 48, 2109 Aldebert Rd., Cleveland, OH 44106-4954, USA; dmk121@case.edu; 2 School of Biological and Population Sciences, Nutrition and Exercise Science, Oregon State University, 103 Milam Hall, Corvallis, OR 97331, USA; melinda.manore@oregonstate.edu

**Keywords:** energy availability, bone health, amenorrhea, sports performance, disordered eating, active females

## Abstract

Female athletes who follow a diet that fails to meet energy and nutrient needs are at risk for musculoskeletal injuries, menstrual disturbances, and poor sports performance. Common nutritional concerns for the female athlete include low energy availability (EA) (i.e., energy intake from food remaining for metabolic processes after accounting for energy expended during exercise) and inadequate dietary intakes (i.e., not meeting sports nutrition guidelines) of carbohydrates, protein, essential fatty acids (EFAs), B-vitamins, calcium, iron, and vitamin D. Low EA and the associated nutrient deficiencies are more common in athletes who compete in weight-sensitive sports (i.e., aesthetic, gravitational, and weight category sports) because low body fat and mass confer a competitive advantage. Other athletes at risk for energy and nutrient deficits include athletes following a vegetarian or gluten-free diet (GFD). Careful dietary planning can help an athlete meet energy and nutrient needs. This review covers the nutrition issues associated with low EA and special diets (i.e., vegetarian and GFD) and describes strategies to help female athletes meet their energy and nutrient needs.

## 1. Introduction

Evidenced-based sports nutrition guidelines provide the foundation for developing diet patterns that support the recovery and training needs of active woman. Athletes, however, may restrict dietary energy intake (EI; kcal/day) to achieve or maintain a low body weight or body fat content to optimize sports performance [1]. In addition, special diets, such as vegetarian and gluten-free diets (GFD), have grown in popularity among athletes related to the belief that they confer health and weight management benefits compared to a more typical diet [2,3,4]. Religious, social, and environmental concerns are other reasons that female athletes choose to eliminate foods or food groups from their diets [2,3,4]. Athletes with inadequate EI and/or who exclude certain foods or food groups may not meet sports nutrition guidelines for key nutrients such as carbohydrates, protein, essential fatty acids (EFAs), calcium, iron, vitamin D, and the B-vitamins [5,6,7,8,9]. As a result, female athletes are at increased risk for musculoskeletal injuries, iron-deficiency anemia, menstrual disturbances, hormonal imbalances, and immune suppression [6,10,11]. In this review, low energy availability (EA), which arises when active females do not meet their energy needs, and the associated health and nutrition consequences are described. Then, the impact of vegetarian diets and GFDs are discussed, and their impact on health and sports performance in athletes are examined. Finally, this review concludes with dietary strategies to improve EA and address associated nutritional deficiencies.

## 2. Methods

Articles were located using an online search of journal databases including EBSCO host, Google Scholar, PubMed, and Web of Science. The key terms used in the article search included female OR women AND athletes OR exercise AND energy availability, energy intake, diet, dietary carbohydrates, dietary protein, dietary fats, essential fatty acids, celiac disease, glutens, gluten-free diet, vegetarian, vegan, dietary iron, iron deficiency, micronutrients, vitamin D, OR vitamin B complex. In addition, a search of key authors was conducted. Finally, articles cited in the bibliography of position stands, and review articles were examined. 

## 3. Low Energy Availability (EA)

Athletes can reduce the risk of injuries and illness while maximizing athletic performance by consuming a diet sufficient in energy (kcal/day) and nutrients to support their exercise training and lifestyle needs [6,9]. Low EA occurs when EI is inadequate to meet the energy demands of exercise and activities of daily living. In this section, EA and low EA are defined followed by a discussion of health and nutritional concerns with low EA.

### 3.1. Definitions

In 2007, low EA was identified as a component of the female athlete triad contributing to low bone mineral density (BMD) and menstrual disturbances [11]. In Table 1, EA and other energy terminology commonly used in sports nutrition are presented. EA is calculated by subtracting exercise energy expenditure (EEE) from total EI and considered to be optimal at >45 kcal/kg fat-free mass (FFM) [11]. Low EA is defined as <30 kcal/kg FFM and has been associated with health issues such as increased risk for stress fractures, low BMD, menstrual dysfunction, hormonal alterations, and immune suppression [6,11]. However, there are issues associated with estimating and using EA in practice, such as the need for equipment to accurately measure resting metabolic rate (RMR) and EEE, an ambiguous definition of physical activities that should be included in the calculation of EEE, and potential underreporting of EI [12,13]. In addition, EA has also been observed to be similar among athletes despite their menstrual status [14,15] with health issues occurring in some athletes with suboptimal levels of EA [11]. Despite these concerns, EA can be estimated utilizing 24-hour recalls or dietary logs for EI, Harris-Benedict or Cunningham for RMR [16,17,18], and factorial equations and metabolic equivalents (METs) for energy expenditure [19]. MET is the ratio of work metabolic rate to RMR of 1.0 kcal/kg/hour where RMR represents metabolic rate while sitting quietly [19,20]. For EA, physical activities with METs >4.0 are recommended to include in EEE calculations [12]. If an athlete is not purposefully dieting, underreporting of typical food intake can be assessed as described by Goldberg et al. [21]. Athletes with estimated EA of <45 kcal/kg FFM should be further evaluated for potential energy and nutrient deficits and educated on the importance of meeting energy needs. 

Low or sub-optimal EA in athletes may be the result of disordered eating, restricted EI to meet weight goals, or the inability to match EI to EEE [5,24,25]. Each of these potential causes are briefly discussed below:

- **Disordered eating**. Disordered eating refers to a range of abnormal behaviors such as dieting, binging, purging, fasting, and excessive exercising. The prevalence of disordered eating is particularly high in weight-sensitive sports (e.g., running, cycling, and wrestling), with ~30%–40% of participants estimated to exhibit disordered eating [1]. As disordered eating behaviors increase in frequency and duration, the athlete may be classified as having a clinical eating disorder (i.e., anorexia nervosa, bulimia nervosa, binge eating disorder, or other specified feeding or eating disorder (OSFED)) [26].
- **Energy Restriction.** Athletes may restrict EI to obtain a certain body weight or body fat level, which is perceived to confer a competitive advantage. Restricting EI puts an athlete at risk for low EA and is more prevalent in athletes who compete in weight-sensitive sports [1]. These sports include gravitational sports (e.g., long-distance running, cycling, and ski jumping), aesthetically judged sports (e.g., gymnastics, diving, and figure skating), and sports with weight classes (e.g., judo, competitive weight lifting, and lightweight rowing) [1].
- **Suboptimal Energy Intake.** Some athletes may not intentionally restrict EI, but consume a high-fiber, low-energy dense diet (i.e., high consumption of fruits, vegetables, and whole-grains) that can increase satiety and put them at risk for low EI and EA [14,25,27,28,29]. In addition, high-intensity exercise may also suppress appetite in female athletes leading to insufficient EI after exercise [30].

In athletes with sub-optimal EA, the registered dietitian nutritionist (RDN) with training in sport nutrition should identify the etiology of low EA after consulting with the athlete’s health care team (e.g., coaches, athletic trainers, and team physicians) to determine if there are disordered eating issues or if EI is being restricted to make weight. Female athletes with suboptimal EI due to high-fiber, low energy dense diets and/or exercise-suppressed appetite should include more energy-dense snacks and foods in their diet (see discussion below) and not rely on hunger as a cue for energy needs [14,25,30]. 

### 3.2. Low EA and Health

Low EA is associated with several health issues in both male and female athletes, such as hormonal disruptions, impaired musculoskeletal health, immune suppression, mood disturbances, and endothelial dysfunction [6]. For female athletes with low EA, a key concern is menstrual dysfunction with reported incidence ranging from 0–60% of active women [31]. Menstrual disturbances may occur as a result of suppressed leptin levels, which disrupt the signaling cascade for estrogen and progesterone synthesis [11]. Specifically, low leptin levels impair gonadotropin releasing hormone (GnRH) pulsatility leading to decreased luteinizing hormone (LH) secretion from the pituitary and finally reduced gonadal production of estrogen and progesterone [32]. The net result is menstrual disruption that occurs along a continuum from mild (e.g., failure to ovulate or luteal phase deficiencies) to severe irregularities (i.e., oligomenorrhea (cycles ≥ 35 days) that may progress to amenorrhea (no menses for >90 days) [11,32]. 

Compromised bone health (i.e., low BMD, stress fractures, and osteoporosis) has also been associated with both low EA and menstrual dysfunction [6,11,33]. The definition for low BMD in premenopausal women is 2.0–2.5 standard deviations (SD) below the mean of BMD of young adults, with osteoporosis defined as >2.5 SD below mean [11]. Athletes typically have a BMD 5%–15% higher than non-athletes and thus may be at risk for poor bone health if BMD >1.0 SD below mean [11]. In female athletes with menstrual dysfunction, low BMD and osteoporosis have been observed to occur in 20%–50% and 10%–13%, respectively [34]. Stress fractures have also been linked to menstrual dysfunction [6,11] with more young amenorrheic athletes (age = 14–25 year, ~61% runners) reporting a history of stress fractures than eumenorrheic athletes (32% vs. 5.9%, respectively) [35]. Beyond bone and menstrual health, low EA has also been associated with metabolic hormonal abnormalities (i.e., low leptin, thyroid, and insulin-like growth factor-1 (IGF-1) levels; elevated cortisol levels) and alterations in skeletal muscle, cardiovascular, central nervous system, immune, and renal function [6,36]. These health risks have been address in previous review articles [1,5,6,9,11,37,38]. 

### 3.3. Nutritional and Sports Performance Concerns with Low EA

Low EA increases the risk for certain nutritional deficiencies, including energy, carbohydrates, protein, EFAs, and micronutrients (i.e., B-vitamins, vitamin D, calcium, and iron) [5,6]. For female athletes, the recommended macronutrient intake ranges from 3–10 g/kg of athlete’s body weight (BW) per day for carbohydrates, 1.2 to 2.0 g/kg BW/day for protein, and 20%–35% of total kcal/day for fat [9,23]. Athletes with low EA often meet their macronutrient needs with carbohydrate intakes at the low end of the targeted range [9,14,39]. However, athletes with low EA who fail to consume adequate levels of carbohydrates or high-quality protein may suffer from impaired glycogen and muscle protein synthesis and bone remodeling [6,9]. Other concerns include inadequate intake of the EFAs, docosahexaenoic acid (DHA) and eicosapentaenoic acid (EPA), which can lead to increased inflammation and oxidative stress and decreased gut calcium absorption further impacting musculoskeletal health [40,41]. Therefore, both the quantity and quality of macronutrient intakes are important to support health and performance in athletes. 

Recovery from exercise and bone health may also be impaired if an athlete is low in key micronutrients such as calcium, iron, vitamin D, and the B-vitamins. Only two studies have examined micronutrient deficiencies in female athletes with low EA (Table 2). Most athletes were reported to have inadequate dietary vitamin D intakes [14,39]. This is not a concern for athletes, who train and live below latitudes of 40°N but is a concern for those living above this latitude [42]. The studies by Viner et al. in cyclists [39] and Cialdella-Kam et al. in female endurance athletes [14] took place in Colorado Springs, CO (latitude of ~39° N) and Corvallis, OR (latitude of ~45° N), respectively. The majority of cyclists also had inadequate dietary intakes of calcium and folate; however, most met dietary recommendations for vitamins B6 and B12 (Table 2) [39]. Reported dietary intakes of calcium, folate, and vitamin B12 were sufficient in the majority of female endurance athletes (Table 2) [14]. Blood levels of vitamins D and B12, folate, and iron were also assessed with no differences between women with exercise-induced menstrual dysfunction (~38% reported taking multivitamin/mineral) and eumenorrheic women (~40% reported taking multivitamin/mineral) [14]. Furthermore, deficiencies were found in only a few athletes (i.e., low serum ferritin levels in three athletes, and iron deficiency anemia in one athlete) [14]. At baseline, most women in this studies had suboptimal EA levels (mean EA = ~35 kcal/kg FFM for both groups), and thus may account for the lower incidences of observed inadequate nutrient intake compared to the cyclists [14,39]. Based on these two studies, nutrient deficiencies can occur in athletes with sub-optimal and low EA; however, more research is warranted.

## 4. Special Diets: Vegetarian and Gluten-Free Diets

Special diets, such as vegetarian diets and GFDs, have increased in popularity among athletes related to perceived health benefits, religious reasons, and environmental and social concerns [2,3,4]. In this section, vegetarian and gluten-free diets in athletes are discussed.

### 4.1. Definitions

Athletes choose to follow specific dietary regimes (e.g., vegetarian or GFD) to meet health and weight goals, reduce gastrointestinal distress, and ultimately improve sports performance, and/or for ethical or financial reasons [2,3,4,9]. The different types of vegetarian diets are defined in Table 3, highlighting key nutrient concerns. To our knowledge, the prevalence of vegetarianism in athletes has only been examined in one study [43]. Based on data collected at the Delhi 2010 Commonwealth Games, approximately one-third of all athletes (n = 351) reported being vegetarian or meat avoiders [43]. Those athletes most likely to adhere to a vegetarian or vegan diet included non-Western athletes (prevalence = 31%), those participating in a weight category sport (prevalence = 42%), and female athletes (prevalence not reported) [43]. Vegetarian athletes were associated with avoiding foods that contained additives and wheat, and cited nutrient composition as the main reason for following a vegetarian diet [43]. As shown in Table 3, athletes can successfully follow a vegetarian diet with carefully planning to ensure adequate intake of energy, carbohydrate, protein, and nutrients. 

A GFD eliminates cereals and products (i.e., wheat, rye, barley, spelt, triticale, kamut, and farina) that contain the wheat protein, gluten. These diets are recommended by health professionals for individuals with celiac disease or non-celiac gluten sensitivity [44,45]. To our knowledge, Lis et al. [3] is the only study examining the prevalence of GFDs in athletes. Athletes (n = 910; 58% female) surveyed ranged from recreational to Olympic medalists, with approximately 66% reporting being endurance athletes [3]. A GFD was reported to be followed by ~20% (n = ~188) of the athletes 90%–100% of the time and by ~70% (n = 262) of endurance athletes >50% of the time [3]. Athletes who adhered to a GFD >50% of the time did so based primarily on self-diagnosis (~57%) of a gluten intolerance, with a majority (~81%) reporting improvement of symptoms attributed to gluten (i.e., abdominal bloating, gas, diarrhea, and fatigue) [3]. Of these, only 9.9% (n = 37) had a clinical diagnosis, and 0.5% (n = 2) did it based on nutritionist/RDN recommendation [3]. A GFD can put an athlete at risk for low intakes of protein and micronutrient deficiencies (i.e., B-vitamins, calcium, vitamin D, iron, and potassium) [46,47,48]. Thus, athletes should determine if a GFD is medically warranted by consulting with a RDN who is part of their health care team. The health benefits and concerns of vegetarian and GFD are explored in the next section. 

### 4.2. Vegetarian and Gluten-Free Diets and Health

Health benefits have been linked to vegetarian diets; however, the health benefits of a GFD in individuals have not been examined. Vegetarian diets have been associated with lower body mass index (BMI) and decreased risk of chronic disease (i.e., hypertension, type II diabetes, metabolic syndrome, and some cancers) [2,49]. However, exercise independently has also been associated with the same health benefits [50]. To our knowledge, the impact of potential synergistic or additive benefits of following both a physically active lifestyle and a vegetarian diet have not been examined. A well-planned vegetarian diet does include a high consumption of fruits and vegetables that provide dietary fiber and anti-inflammatory and immune-supporting micronutrients and phytochemicals. 

A GFD is prescribed for those with celiac disease [45] or non-celiac gluten sensitivity [51]. The impact of a GFD on body weight is equivocal. In both normal and overweight celiac patients who adhered to a strict GFD for 2 years, weight gain was observed [52]. Conversely, a GFD has also been associated with improved BMI (i.e., weight gain in underweight and weight loss in overweight patients) in celiac patients [53]. Research is lacking on the impact of a GFD on body mass and composition in athletes. In the next section, potential nutrient deficiencies and implications on sports performance of a GFD are discussed.

### 4.3. Nutritional and Sports Performance Concerns with Special Diets

Any diet that restricts certain foods or food groups can increase the risk of low EA and deficiencies in key nutrients such as protein, carbohydrates, EFAs, and micronutrients (i.e., B-vitamins, calcium, vitamin D, and iron). The athlete consuming a vegetarian diet often consumes more fruits and vegetables than the non-vegetarian athlete adding many nutritional benefits (i.e., providing phytochemicals and antioxidants to support exercise training and health) but some disadvantages as well (Table 3). One key nutritional concern is the high fiber content of the vegetarian diet, which may lead to early satiety and appetite blunting, reducing EI and contributing to low EA [14,25,27,28,29]. Vitamin D is a concern for athletes who live in latitudes >40°N as many athletes fail to meet recommendations from diet alone. In a systemic review and meta-analysis, Farrokhyar et al. [42] reported a high prevalence (~66%) of vitamin D deficiency among athletes, particularly in those who participated in indoor sports (i.e., swimming, basketball, dancing, gymnastics, volleyball, and wrestling) [42]. Good dietary sources of vitamin D include fatty fish and fortified products such as dairy products, cereals, and orange juice [54]. Thus, athletes who do not consume dairy products and/or fish may be at increased risk for vitamin D deficiency. For these individuals, a biochemical assessment of vitamin D status is warranted. Finally, a vegetarian diet can provide adequate protein to support exercise training if the athlete has adequate EI [8,55]. Thus, the concerns for nutrient deficiencies for vegetarian athletes are similar to those discussed above for athletes with low EA (i.e., carbohydrates, protein, EFAs, and certain micronutrients). Sports performance, however, has not been evaluated in vegetarian athletes [9]. A well-planned diet should be able to support the training and health needs of the vegetarian athlete; please refer to “Vegetarian Sports Nutrition” for more information [2]. 

Nutrient deficiency in those consuming GFDs has not been well examined. However, Kulai and Rashid [47] examined the nutritional adequacy of gluten-free food products (n = 71) available in Canadian stores. Mean protein content of gluten-free products was lower for both bread (mean difference = 4 g/100 g of bread) and pasta (mean difference = 5.8 g/100 g of pasta) vs. regular products [47]. Additionally, gluten-free breads contained less iron and more total fat than regular bread; however, saturated fat did not differ [47]. For pasta, gluten-free products had higher carbohydrate content and lower fiber, sugar, iron and folate content [47]. In addition, the price (per 100 g of product) was ~1.6-folds higher for gluten-free products compared to regular products [47]. Similarly, Wu et al. [46] and Mishbach et al. [48] compared the nutrient content of gluten-free to gluten-containing food products in Australia and Austria, respectively. Consistent with Kulai and Rashid [47], mean protein content was lower in gluten-free product vs. regular products [46,48]. However, there were no differences in total energy, total carbohydrates or sugars, total or saturated fat, or sodium levels [46,48]. Contrary to Kulai and Rashid [47], mean fiber content was higher in gluten-free pasta and bread but lower in ready-to-eat cereals vs. gluten-containing Australian products [46] and no different in Austrian products [48]. In addition, iron content did not differ in Austrian products [48], but Wu et al [46] did not report the iron content of their gluten-free products. However, consistent with Kulai and Rashid [47], Austrian gluten-free products had a 1.7-fold higher cost than regular products [48]. In conclusion, gluten-free products are costlier and have lower protein content than regular products, with mixed findings on differences in iron and fiber content of these foods [46,47,48]. 

To our knowledge, the impact of GFDs on exercise performance has only been examined in one study [56]. Lis et al. [56] had non-celiac cyclists (n = 13) follow either a GFD or regular diet for seven days in a double-blind, placebo-controlled, crossover design study. No observed difference was found in time trial performance (i.e., 15 min cycling time trial after completion of 45 min steady state cycling at 75% W_max_), inflammatory response, GI symptoms, or overall well-being. Thus, for the non-celiac athlete, GFD may not confer any advantages in sport performance [56]. However, GFDs are recommended for athletes with celiac disease [45] or non-celiac gluten sensitivity [51]. An example of a one-day GFD menu is provided in Table 4. Athletes without a diagnosed condition should consult a RDN to discuss the pros and cons of such a diet and to ensure both energy and nutrient needs for sports performance are met. 

## 5. Nutritional Strategies for Low EA

The reversal of low EA in a female athlete first requires the identification of the underlying causes of low EA and then counseling and education on the importance of meeting energy needs. Athletes with eating disorders require the engagement of a healthcare professional team trained in eating disorders, including RDN, athletic trainer, coach, psychiatrist, and primary care physician [57]. Successful strategies to improve low EA are increasing EI by adding energy dense snacks to the diet, not skipping meals, and/or decreasing EEE (i.e., reduce training volume by including a day off) [5]. For many athletes, an improvement of EA by ~230 to 380 kcal/day appears to be adequate to restore menstrual status in conjunction with weight gain of 2–5 kg [14,58,59,60]. A successful strategy is to incorporate energy dense snacks and foods that are rich in nutrients, as presented in Table 5. In addition, adequate intake of the bone-building nutrients (i.e. calcium, 1000 mg/day and vitamin D, 600 IU/D) is vital. Assessment of B-vitamin and iron status should also be done. Finally, diet and exercise interventions alone may not be sufficient to restore bone health and/or menstrual status [58,59]; thus, it is important that the health care team be engaged in the athlete’s care. 

## 6. Conclusions

From a diet perspective, the key nutritional concern for female athletes remains meeting the energy demands of their training. Restricted-energy diets or special diets, such as vegetarian diets or GFDs, put an athlete at risk for low EA and associated musculoskeletal, hormonal, and other health concerns. The underlying cause of low EA must first be identified to determine a successful treatment approach. In athletes with low EA related to inadvertent causes (i.e., early satiety or appetite suppression), a successful approach is to incorporate energy dense snacks and foods into their diet. Vegetarian diets and GFDs can meet the energy and nutrient needs of the athlete with careful dietary planning. Athletes should refer to an RDN who specializes in sport nutrition for additional guidance on implementing these diets. 

## Figures and Tables

**Table 1:** Energy terminology [9,11,22,23].

Terminology	Definition
Total Energy Expenditure (TEE; kcal/day)	Total daily energy expended for metabolic processes, daily living activities, and exercise. TEE is the sum of basal metabolic rate, thermic effect of food, and thermic effect of activity.
Total Energy Intake (EI; kcal/day)	Total daily energy obtained from food, fluids, and supplements.
Energy Balance (EB; kcal/day)	The energy difference between EI and TEE. Positive EB is associated with weight gain and negative EB with weight loss.
Basal Metabolic Rate (BMR; kcal/day)	Total energy used at rest for organ functions, body temperature regulation, and cellular functions. Typically measured after an overnight stay in a metabolic ward.
Resting Metabolic Rate (RMR; kcal/day)	Total energy used for organ functions, body temperature regulation, and cellular functions in relaxed state. The difference between BMR and RMR is the conditions in which the measurement is taken, with estimates of RMR up to ~10% higher than BMR.
Thermic Effect of Activity (TEA; kcal/day)	Energy expended during exercise and daily living activities.
Thermic Effect of Food (TEF; kcal/day)	Energy consumed above BMR for the digestion, absorption, and storage of nutrients and accounts for ~10% of TEE.
Exercise Energy Expenditure (EEE; kcal/day)	Energy expended during exercise.
Energy Availability (EA; kcal/day)	Energy remaining for all biological process after accounting for the energy expended during exercise.

**Table 2:** Reported prevalence of dietary micronutrient insufficiency in athletes with low energy availability (EA).

Study	Athletes Examined	Diet Method	Nutrient	Prevalence of Low Intake	Reference Value ^3^
Cialdella-Kam et al., 2014 [14] ^1^	Female Endurance Athletes: -Eumenorrheic athletes (n = 9) -Athletes with exercise-induced menstrual dysfunction (n = 8)	7-day weighed food records	Calcium:	0%	800 mg/day
			Vitamin D:	>50%	400 IU/day
			Folate:	24%	320 mcg DFE
			Vitamin B6:	-	-
			Vitamin B12:	12%	2.0 mcg/day
Viner et al., 2015 [40] ^2^	Cyclists: -Male Cyclists (n = 6) -Female Cyclists (n = 4)	3-day food records ^4^	Calcium:	75%	1000 mg/day
			Vitamin D:	100%	600 IU/day
			Folate:	90%	400 mcg DFE
			Vitamin B6:	20%	1.3 mg/day
			Vitamin B12:	20%	2.4 mcg/day

^1^ Dietary intake was compared to estimated average requirement (EAR). Prevalence of vitamin B6 insufficiency was not reported; ^2^ Dietary intake was compared to recommended dietary allowance (RDA); ^3^ DFE = dietary folate equivalents, IU = international units; ^4^ Participants received a handout for visual estimates of food portions and were encouraged to use a scale or household measure for quantification of foods.

**Table 3:** Key nutrient concerns for athletes following vegetarian diets [2,4,7,8,9].

Types of Vegetarian Diets	Essential Fatty Acids	Vitamin B-12	Vitamin D	Calcium	Iron	Zinc
**Semi-vegetarian or “Flexitarian”:** A vegan diet with occasional meat and dairy consumption			X		X	
**Pescatarian:** A diet that excludes meat and poultry and includes fish, diary, and eggs.			X		X	
**Lacto-ovo Vegetarian:** A diet that excludes: meat, fish, and poultry and includes eggs and dairy.			X		X	
**Vegan:** A diet that excludes meat, fish, poultry, eggs, and dairy	X	X	X	X	X	X

“X” indicates that diet may increase risk of nutrient deficiency.

**Table 4:** Example gluten-free diet menu for a female athlete ^1^.

Meal	Energy (kcal)	Carbohydrate (g)	Protein (g)	Fat (g)
Breakfast	550	100	15	10
Orange juice (8 fl oz)				
Yogurt, low-fat, plain (6 oz)				
Honey (1 tbsp)				
Granola, gluten free (2 tbsp)				
Toast, gluten-free (2 slice)				
Butter (1 pat)				
Morning Snack	360	50	7	15
Apricot, dried (1/2 cup)				
Almonds, roasted (1 oz)				
Lunch	685	85	30	25
Salmon (3 oz)				
Baked medium sweet potato with 1 pat of butter and 1 tsp of brown sugar				
Salad (mixed greens, red peppers, cucumber, green onions)				
Oil and vinegar (1 tbsp)				
Broccoli, cooked (1 cup)				
Dark chocolate (1 oz)				
Afternoon Snack	290	70	3	0
Fruit smoothie (15 fl oz)				
Dinner	970	130	45	30
Chicken breast, grilled (3 oz)				
Wild rice, cooked (1 cup)				
Vegetable stir fry with oil (1 cup)				
Corn on the cob (1 ear)				
Milk, 1% (1 cup)				
Chocolate chip cookies, gluten-free (2 each)				
Total	2855	435	100	80

^1^ This menu provides ~2800 calories; 6.7 g/kg of body weight (BW) of carbohydrates, 1.5 g/kg BW of protein, and 26% of total kcal of fat for a 65 kg female athlete.

**Table 5:** Energy dense snacks ^1^.

Snack Ideas	Serving Size	Energy (kcal)	Carbohydrate (g)	Protein (g)	Fat (g)
Peanut butter on whole wheat crackers	Peanut butter = 2 Tbsp (28 g)	308	27	10	20
	Crackers = 6 each (28 g)				
Low-fat strawberry Greek yogurt	5.3 oz (150 g)	120	18	12	4
Part-skim mozzarrella cheese on whole-wheat pita	Cheese = 1 oz (28 g)	145	16	10	5
	Pita = 4“ diameter (28 g)				
Hummus with low-fat, baked tortilla chips	Hummus = 4 Tbsp (60 g)	228	31	8	7
	Chips = 1 oz (28 g)				
Strawberry banana smoothie with ice and low-fat yogurt	12 fl oz (347 g)	226	52	3	<1

^1^ Energy (kcal) and macronutrients (g) represent means provided in US Department of Agriculture, Agricultural Research Service, Nutrient Data Laboratory. USDA National Nutrient Database for Standard Reference, Release 28. Version Current: September 2015, slightly revised May 2016 (https://ndb.nal.usda.gov/ndb/).
